# Editorial: Advances in Goal, Plan and Activity Recognition

**DOI:** 10.3389/frai.2022.861669

**Published:** 2022-03-16

**Authors:** Mor Vered, Reuth Mirsky, Ramon Fraga Pereira, Felipe Meneguzzi

**Affiliations:** ^1^Department of Data Science and AI, Monash University, Melbourne, VIC, Australia; ^2^Deparment of Computer Science, Bar Ilan University, Ramat Gan, Israel; ^3^Dipartimento di Ingegneria Informatica, Automatica e Gestionale (DIAG), Sapienza Universitá di Roma, Rome, Italy; ^4^Deparment of Computer Science, University of Aberdeen, Aberdeen, United Kingdom

**Keywords:** goal recognition, plan recognition, behavior recognition, action recognition, plan recognition as planning, plan libraries

We are living in a wonderful era of exciting technological advancements. Prominent among these advances is the ever expanding reach of Artificial Intelligence (AI) and Machine Learning approaches. These enable us to achieve superior natural language understanding (Allen, 1988; Devlin et al., 2018), tailored recommendation systems (Malik et al., 2020), efficient personal assistants (de Barcelos Silva et al., 2020), accessible service robots (Veloso et al., 2015) and brings us to the brink of making autonomous vehicles a reality (Kuutti et al., 2020). However, to fully maximize the potential of these technological advancements, these systems must be able to perform efficient and dynamic *goal, plan*, and *activity recognition*.

*Goal Recognition* refers to the ability to recognize the intent of an acting agent through a sequence of observed actions in an environment (Meneguzzi and Pereira, 2021). In most cases, this recognition takes place prior to the fulfillment of the intent, which requires anticipating the future by predicting the acting agent's coming moves through an understanding of its internal reasoning. People are particularly gifted in this domain, as they are able to understand the intent implied behind obscure words, abstract hand gestures, initial sketches, and more (Grice, 1975; Levelt, 1993). It is this ability that makes us great communicators and allows us to work together cohesively as a team when needed.

*Plan Recognition* is a closely related problem to goal recognition, where the premise of the observer is to recognize *how* the acting agent intends to reach its goal. *Goal and Plan Recognition* are two interdisciplinary AI problems, originally defined by Kautz and Allen (1986). These problems can be addressed using techniques from automated planning, natural language understanding, psychology, human-computer interfaces, machine learning, and more (Mirsky et al., 2021). However, many of these techniques cannot be trivially applied to the problem of goal and plan recognition. For example, when leveraging automated planning for goal recognition, the recognition task requires significantly more information than the analogue planning task, as it includes the knowledge of the observing agent, and the belief about the knowledge of the acting agent. Representing this knowledge can include various limitations on the observations such as partialness and noise, correctness, partial domain model, and missing knowledge of an acting agent's preferences (Meneguzzi and Pereira, 2021). As AI systems become more prevalent, new challenges arise to create accurate, explainable, and robust methods for goal and plan recognition algorithms in the real world.

Research on goal and plan recognition has seen substantial recent research activity. Efforts include relaxing some assumptions about the underlying recognition problems, dealing with domain models with imperfections and spurious observations, expanding recognition to continuous and stochastic domains, as well as those populated by multiple agents (Masters and Vered, 2021). In the current compilation, authors contribute to the scope of goal and plan recognition research, challenging current capabilities and expanding the boundaries of the field.

Carlson et al. introduce a novel application of the multinomial Hidden Markov Model (HMM) technique to predict the behavior of potentially hostile naval vessels and show promising results on simulated data. The authors achieve this through the modeling of the rate of change as observed symbols and the mapping of observations to intent through an HMM. Rabkina et al. develop a new goal recognition system building on the Analogical Theory of Mind (AToM) model and analogy-based reasoning. This approach complements current research by focusing on the high inspectability of the observed agent's mental model and dealing with unreliable observations and uninspectable agents. The authors instantiate the approach in two experimental domains, a Minecraft based domain and Monroe, an important domain based on disaster management scenarios and also contrasted with other state-of-the-art recognition techniques.

Fitzpatrick et al. tackle the problem of recognizing the complex behavior of a team member in the context of the real time, continuous environment of aerial combat. Observations within this domain necessarily have high state space dimensionality to correctly portray the setting; including position, orientation, linear velocity and acceleration. Recognition calls for rapid responses to changing conditions and the frequent absence of reliable data. To do this Fitzpatrick et, al. extend upon a state-of-the-art recognition approach (*Mirroring*, Vered and Kaminka, 2017) and alter it to enable recognition of complex, continuous, behaviors.

Mirsky et al. provide a standardized framework to evaluate plan-library based recognition algorithms. This work provides a comparison of two common plan recognition algorithms (Avrahami-Zilberbrand and Kaminka, 2005; Geib and Goldman, 2009) and analyzes their properties theoretically and empirically using the new framework. Such evaluations can provide useful insights that will help the recognition community achieve improved performance for their algorithms, as exemplified by the new hybrid algorithm presented at the end of the paper.

Weerawardhana et al. introduce a new class of problems tightly related to plan recognition: online intervention problems, where the challenge of the observer is to decide when to intervene in order to help a user. This work introduces several types of intervention problems and proposes solutions that combine plan recognition techniques with classification algorithms. The authors extend the proposed models to be able to perform human-aware intervention, which is evaluated on a real-world domain where it outperforms common plan recognition algorithms.

Masters et al. introduce the Extended Goal Recognition (XGR) framework for goal recognition with the specific goal of capturing certain properties of human behavior and its subsequent recognition. This enables them to describe deliberately deceptive behavior, as well as the counterpart in goal recognition of being able to be deceived by such behavior. The authors illustrate the various ways in which deception can occur in the model through examples from stage magic and how magicians use their understanding of the expectations of other humans when recognizing behavior to deceive them.

And finally, Treger and Kaminka challenge existing work (Masters and Sardina, 2019) by hypothesizing that humans prioritize recognizing known plans and familiar goals. The authors develop a novel plan-library based approach to goal recognition, called Library-based Rational Goal Recognition (LRGR) which combines rationality-based and plan-library based techniques for recognizing goals and is able of coping with both complete and incomplete plan libraries, generating new plans when existing ones do not match with the observations.

## Author Contributions

All authors listed have made a substantial, direct, and intellectual contribution to the work and approved it for publication.

## Conflict of Interest

The authors declare that the research was conducted in the absence of any commercial or financial relationships that could be construed as a potential conflict of interest.

## Publisher's Note

All claims expressed in this article are solely those of the authors and do not necessarily represent those of their affiliated organizations, or those of the publisher, the editors and the reviewers. Any product that may be evaluated in this article, or claim that may be made by its manufacturer, is not guaranteed or endorsed by the publisher.

## References

[B1] Allen J. (1988). Natural Language Understanding. Benjamin-Cummings Publishing Co., Inc.

[B2] Avrahami-Zilberbrand D. Kaminka G. A. (2005). Fast and complete symbolic plan recognition, in IJCAI, 653–658.

[B3] de Barcelos Silva A. Gomes M. M. da Costa C. A. da Rosa Righi R. Barbosa J. L. V. Pessin G.. (2020). Intelligent personal assistants: a systematic literature review. Expert Syst. Appl. 147, 113193. 10.1016/j.eswa.2020.113193

[B4] Devlin J. Chang M.-W. Lee K. Toutanova K. (2018). Bert: pre-training of deep bidirectional transformers for language understanding. arXiv preprint arXiv:1810.04805.

[B5] Geib C. W. Goldman R. P. (2009). A probabilistic plan recognition algorithm based on plan tree grammars. Artif. Intell. 173, 1101–1132. 10.1016/j.artint.2009.01.003

[B6] Grice H. P. (1975). Logic and conversation, in Speech Acts (Brill), 41–58.

[B7] Kautz H. A. Allen J. F. (1986). Generalized plan recognition, in AAAI, Vol. 86, page 5.

[B8] Kuutti S. Bowden R. Jin Y. Barber P. Fallah S. (2020). A survey of deep learning applications to autonomous vehicle control. IEEE Transa. Intell. Transport. Syst. 22, 712–733. 10.1109/TITS.2019.2962338

[B9] Levelt W. J. (1993). Speaking: From Intention to Articulation, Vol. 1. MIT Press.

[B10] Malik S. Rana A. Bansal M. (2020). A survey of recommendation systems. Inf. Resour. Manage. J. 33, 53–73. 10.4018/IRMJ.2020100104

[B11] Masters P. Sardina S. (2019). Goal recognition for rational and irrational agents, in AAMAS.

[B12] Masters P. Vered M. (2021). What's the context? implicit and explicit assumptions in model-based goal recognition, in Proceedings of the International Joint Conference on Artificial Intelligence (IJCAI), 4516–4523.

[B13] Meneguzzi F. R. Pereira R. F. (2021). A survey on goal recognition as planning, in Proceedings of the 30th International Joint Conference on Artificial Intelligence (IJCAI), 2021, Canadá.

[B14] Mirsky R. Keren S. Geib C. (2021). Introduction to symbolic plan and goal recognition. Synthesis Lect. Artif. Intell. Mach. Learn. 16, 1–190. 10.2200/S01062ED1V01Y202012AIM047

[B15] Veloso M. Biswas J. Coltin B. Rosenthal S. (2015). Cobots: robust symbiotic autonomous mobile service robots, in Twenty-Fourth International Joint Conference on Artificial Intelligence.

[B16] Vered M. Kaminka G. A. (2017). Heuristic online goal recognition in continuous domains. arXiv preprint arXiv:1709.09839.

